# Human Health Risk Assessment on the Consumption of Apples Growing in Urbanized Areas: Case of Kharkiv, Ukraine

**DOI:** 10.3390/ijerph18041504

**Published:** 2021-02-05

**Authors:** Yuliia Medvedeva, Anatolii Kucher, Joanna Lipsa, Maria Hełdak

**Affiliations:** 1 National Scientific Center «Institute for Soil Science and Agrochemistry Research Named after O. N. Sokolovsky», 4, Chaikovska Street, 61024 Kharkiv, Ukraine; julia.ukrkharkiv@gmail.com; 2 Department of Ecology and Neoecology, V. N. Karazin Kharkiv National University, Svobody sq., 6, 61022 Kharkiv, Ukraine; kucher@karazin.ua; 3 Institute of Spatial Management, Wroclaw University of Environmental and Life Sciences, ul. Grunwaldzka 55, 50-357 Wroclaw, Poland; joanna.lipsa@upwr.edu.pl

**Keywords:** urban agriculture, heavy metals, environmental safety, human health, hazard index

## Abstract

This study aims to determine the safety of consumption of plant products grown in Kharkiv, Ukraine. Kharkiv, as well as many other post-Soviet cities, is environmentally characterized by the widespread growing of edible plants—from industrial areas to school gardens—as well as the presence of a significant number of nature management conflicts, the location of heavy industry, the prevalence of obsolete environmentally unfriendly transport, etc. The article presents the results of the study of apple samples taken in different functional zones of Kharkiv city, Ukraine. The results of the study showed that the maximum levels of heavy metals were exceeded in apple samples from all sampling sites: Pb—from 11.47 to 38.86 times; Cd—from 1.76 to 5.68 times (of the norms of the FAO and EU). The most polluted were samples from the residential areas, which is partly due to significant land pollution from various types of waste. Levels of hazard index (HI) differ by age groups: from 24.37 to 70.11 HI (children group, 1–6 years); from 10.28 to 29.59 HI (children group, 7–16 years); from 0.88 to 2.53 HI (adult group, 18–65 years). Non-carcinogenic risks can be related to disorders of the immune system, blood, urinoexcretory, and nervous systems as well as problems in the functioning of liver and kidneys. The total carcinogenic risk of eating apples exceeds the permissible level.

## 1. Introduction

The current stage of humanity development is characterized by high rates of urbanization with increasing urban area and the important place of cities in social life and the world economy. According to the data, provided by the United Nations, in 2020, at least 55% of the world population live in cities, and by 2050, the figure will reach 68% [1]. Cities have become the principal environment of human living.

Almost all kinds of economic activity are concentrated within urban areas. The same situation applies to the agrarian sector, which is characterized by development of the directions of urban agriculture (UA) and peri-urban agriculture (PUA). UA suggests production of agricultural products in the territory of cities: roofs of buildings, household plots or other unoccupied lands.

The active development of UA in recent decades has been related to the issue of food safety. Nowadays, many cities in the world satisfy the majority of personal needs for food by means of UA. In particular, the indices of contribution of UA and PUA in the production of vegetables for consumption by urban residents are equal to 90% in Accra, Ghana; 85% in Shanghai, China; 80% in Mexico, Mexico; 65% in Dakar, Senegal, etc. [2]. Thus, UA is a significant component for provision of the food security of modern cities.

In addition to the food function, UA performs many other functions: improving the quality of atmospheric air by absorbing pollutants, normalizing the microclimate of the urban environment by changing the parameters of radiation balance and evaporation rates, and optimizing technogenic landscapes through green infrastructure [3]. Also, city gardens are becoming part of environmental education in schools, where children interact with nature in the process of caring for plants [4].

Urban agriculture is a topical subject of scientific research today. In previous publications, the authors dealt with state intervention on agricultural land [5], comparing Poland and Ukraine in terms of agricultural development and production scale [6,7]. Recent studies also concerned access to green areas in cities and the way these areas are used [8]. Papers deal with the issues of green areas available to residents and community gardens, which are created in many European countries in response to the need of the city residents for contact with nature and healthier living conditions. They create the possibility of active communing with nature, offer various forms of gardening activities aimed at users of various age, social, and ethnic groups. They enable ecological education, promote a healthy lifestyle, and teach respect for nature. Urban gardening can play an important role in the sustainable development of modern cities.

In agriculture, we speak about sustainable development as the balance of three aspects—economic, ecological, and social [9,10]. These aspects must also function in cities.

However, there is also still discussion on the issue of the safety of vegetable products, grown within the urban area. Plants act as acceptors of anthropogenic geochemical flows in the urban environment, accumulating pollutants from the air, soil, and water. In particular, urban soils are characterized by the formation of technogenic geochemical anomalies caused by the influence of different types of land use—from recreational to industrial.

Heavy metals (HM) are one of the main pollutants of urban geosystems. The results of many scientific studies have indicatet the excess of the geochemical background of heavy metals in urban soils around the world [11,12,13]. The danger of heavy metal contamination of soils lies in their long half-life and increased concentration in the food chain due to biomagnification [14]. The use of food products contaminated with heavy metals is associated with their toxic effects on the human body and the risks of teratogenic, carcinogenic, and mutagenic effects [15].

It is important to note that the increase of the permissible values of HM by up to ten fold was observed in vegetable crops, grown in the territory of the cities of Italy [16], Romania [17], Bangladesh [18], and many other countries. Considering the above, the research on the peculiarities of formation of the chemical content of UA products is an actual issue and of scientific concern.

Results of recent researches confirm the uncertainty among scientists concerning the safety of consumption of UA products. The chemical content of the crops of urban geosystems is characterized by significant differentiation depending on the region of growing, particularly the level of social and economic development and ecological conditions of the territory. In the economically developed countries, the risks from consumption of urban vegetable crops are related with emission of pollutants by industrial enterprises and motor vehicles. In particular, the increased concentrations of HM were found in vegetable products of city gardens in Brazil [19], Korea [20], Spain [21], Germany [22], and many other countries.

In general, one can observe a relation between the location of crops growing within the urban environment and their chemical content. The analysis of the vegetable samples, grown in the city gardens in the territory of the Italian city Bologna, confirms the increased risk of HM accumulation by 1.5 times in the crops, grown at a distance of 10 m from the motor way as compared to the ones, grown at a distance of 60 m [16]. The authors of another study [23], found out significant differences in pollutant concentrations in vegetables, grown in different functional zones of Sao Paulo city, Brazil. Those differences were caused by the intensity of traffic, particularities of weather conditions, and building systems in the different zones of urban environment. High buildings served as barriers against polluted air penetration.

The hazard from consumption of urban vegetable products in countries with a low level of industrial development is mainly related to bacteriological environmental pollution. Agyei and Ensink [24] studied 500 samples of salad from Accra, Ghana. The excess of the content of intestinal bacteria was found above 80% in the chosen samples. Use of sewage water for irrigation on urban farms is one of the main reasons of pollution. A similar problem is specific for Madagascar, where risks of consumption of urban vegetable products are mainly of bacteriological character. An extra hazard factor is the location of land plots of vegetables growing in the lowlands of cities, flooded with sewage water [25].

The monitoring of the environment by functional zones is used to identify the land plots, characterized by a low level of pollution and can be potentially suitable for organization of agroecosystems within cities with unsatisfactory ecological conditions. Naser et al. [26] examined the chemical content of leaf vegetables, grown in one control zone (land plot of the educational research institution) and two industrial zones of Bangladesh. According to the research results, concentrations of the majority of HM in leaf vegetables of the control land plot were lower than the permissible level, set by the FAO and WHO [27]. Higher concentrations of HM were observed in the leafy vegetables of industrial zones.

The way of growing vegetable products is of great importance, i.e., directly in the soil, on soilless systems or with hydroponics. The data of another study, conducted in the Italian city of Bologna, show that cereals, grown on a soilless system, have lower indices of HM content as compared to a soil system, particularly with up to 70% of Cr, up to 61% of Cu, up to 45% of Cd, up to 81% of Ni of the soil system levels [28]. Such an approach is reasonable with a high level of soil cover pollution, requiring the application of expensive technologies for purification.

This study aims to determine the safety of consumption of plant products grown in post-Soviet cities. The problem of ecological safety is crucial for the development of UA. The analysis of recent publications confirms the pollution of vegetable products, grown within urban environments, with pollutants of chemical and bacteriological origin. The polluted samples of vegetable products were found in the city gardens of different regions—from agrarian countries of Africa to economically developed countries of Western Europe. However, spatial regularities of formation of the chemical content of vegetable products of urban geosystems of some regions, including the post-Soviet area, have been little studied. Specific attention should be paid to examination of the peculiarities of pollutant accumulation in the plants, grown on the land plots of different types of land use.

## 2. Materials and Methods

**Figure 1:**
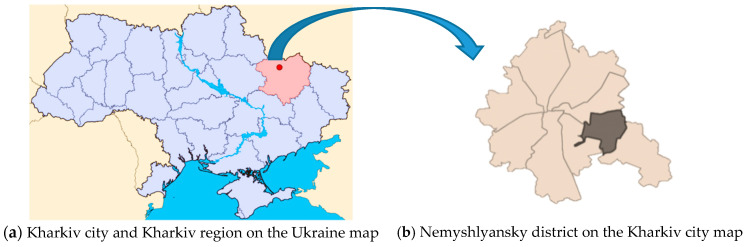
Location of research area (maps from open sources Google).

The research was conducted in the territory of Nemyshlianskyi district of Kharkiv city, Ukraine. The territory has an urban landscape, typical for post-Soviet countries, i.e. combination of industrial, residential, and recreational zones. By the quantitative indices, the population of the district is typical of the medium-size cities of Ukraine and in 2020, it was 140,000 people (Figure 1).

**Figure 2:**
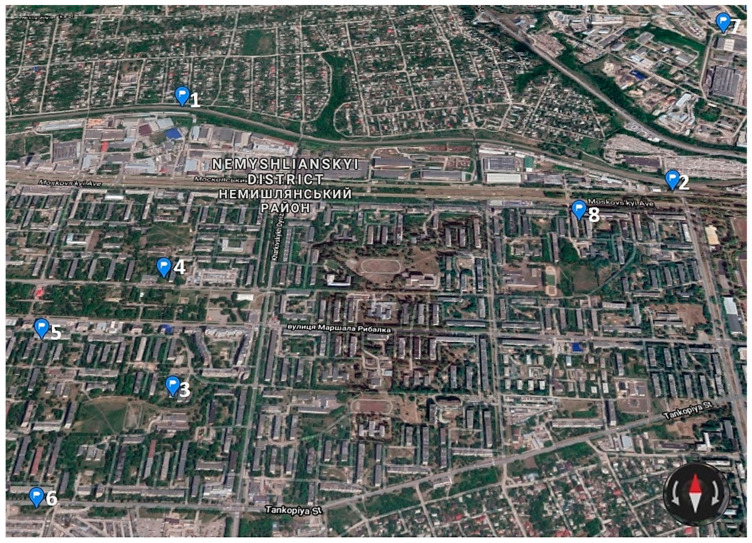
Sampling sites (Ukraine, Kharkiv city, Nemyshlianskyi district). 1—private sector, 2 and 6—transport area, 3—school, 4—square park, 5 and 8—household plots of multi-story apartment buildings, 7—industrial cluster (maps from open sources Google).

The researchers conducted the experiment on apple fruits as it is the most common fruit crop in the city gardens of Ukraine. The apple samples were selected from 8 testing land plots. The points of the sample selection were located within different functional zones of the urban geosystem, i.e. household plots of multi-story apartment buildings, the private sector, a school, a transport area and an industrial cluster (Figure 2).

The selection of apple samples was carried out in accordance with the requirements of the State Standards of Ukraine (DSTU) ISO 874-2002. As to representativeness of the data from each apple tree, the researchers took 10 fruits, which had no visible sign of disease or mechanical damages. The transportation of apple samples to the laboratory took place in plastic bags. The average sample from one sampling point was determined by the quartering method. Sample preparation was carried out in accordance with the requirements of the technical standard GOST 26929-94. To remove the surface dust of the apple samples, they were washed with tap water, supplied to the residential complexes of the district from the city source of the central water supply. The inedible part was removed from the fruits, i.e., seed and stem. The prepared samples were brought to an air-dry state in a drying cupboard with a thermostat.

Chemical analysis of the selected samples was conducted on the basis of the educational and research laboratory of analytical ecological researches of V. N. Karazin Kharkiv National University. The samples of apples were analyzed for content of HM such as Cr, Zn, Cu, Cd, Pb. The content of HM was determined using atomic absorption spectroscopy. Before determining the content of heavy metals, apple samples were mineralized using the dry ashing method, according to DSTU 7670: 2014. Acidic extraction of heavy metals from ash was carried out using an HCI solution in a 1:1 ratio. The solution was prepared in a fume hood. The content of heavy metals was determined using an atomic absorption spectrophotometer С-115М1-ПК. The spectrophotometer has metrological attestation and is listed in the State register of measuring devices of Ukraine (certificate UA-M1/1-135-97).

To estimate the safety of consumption of the studied apple fruits, the researchers calculated the hazard quotient. The hazard quotient is determined as a correlation of the actual concentration of HM and the maximum permissible level in apples by Formula (1) [29]:

(1) $$\mathrm{Hazard}\;\mathrm{ratio}=\frac{\mathrm{Actual}\;\mathrm{content}\;\mathrm{of}\;\mathrm{heavy}\;\mathrm{metals},\mu g/\mathrm{kg}\;}{\mathrm{Maximum}\;\mathrm{levels}\;\mathrm{for}\;\mathrm{heavy}\;\mathrm{metals},\mu g/\mathrm{kg}\;}$$

The figures of actual concentration of HM were obtained according to the results of the physical and chemical analyses of the selected samples (the above-described methodology). The obtained values of the actual concentrations of HM were compared with their maximum levels, set by the FAO [30], EU [27] and the Ministry of Health of Ukraine [31]. According to the mentioned regulatory documents for fresh fruits, maximum levels have been set, particularly Pb—0.1 mg/kg; Cd—0.05 mg/kg (FAO/EU), and Cd—0.03 mg/kg (Ministry of Health of Ukraine). The content of chemical substances or compounds in food products is considered safe for human health in the case where the index of the hazard quotient does not exceed 1.

To estimate safety of consumption of the studied apple fruits for human health, the work presents the calculated non-carcinogenic and carcinogenic risk. According to the Environmental Protection Agency (EPA), the non-carcinogenic hazard quotient (HQ) is the correlation of the potential impact of a substance and the level, at which side-effects are not expected. The HQ level is calculated by the Formulas (2) and (3), adopted for peroral intake of pollutants with vegetable products [32,33]:

(2) $$\mathrm{HQ}=\frac{\mathrm{EDI}}{\mathrm{RfD}},$$

(3) $$\mathrm{EDI}=\frac{\mathrm{DI}\;\times C_{M}}{\mathrm{BW}},$$

where:

RfD (reference dose)—stands for estimation of daily peroral impact on the population, which does not cause harmful effects in the human body during his/her lifetime (Integrated Risk Information System, 2020);

EDI (estimated daily intake)—stands for the value of a peroral daily intake of HM in the human body with apples (μg/kg bw/day);

DI (daily intake)—stands for the daily consumption of apples, kg/day;

C_M_—stands for the concentration of each studied HM in the apples;

BW (bodyweight)—stands for the average weight of a group of people, participating in the experiment.

The total non-carcinogenic risk from all studied chemical elements and compounds from the only way of impact is called the hazard index (HI). According to EPA, the HI is calculated by summing the values of the HQ for each of the studied chemical elements and compounds using Formula (4) [34]:

(4) $${\mathrm{HI}}_{i}=\;\sum {\mathrm{HQ}}_{i}$$

To calculate the HI and HQ, the researchers used the actual values from the EPA-IRIS base: Cd—0.001 mg/kg/day; Zn—0.3 mg/kg/day; Cr—0.003 mg/kg/day [d27]. RfD for Pb and Cu, under peroral intake to the human body with food products, are not mentioned in the IRIS. Thus, RfD for those elements were taken from the data of modern scientific researches, which determined the values of RfD basing on the NOAEL (The no observed adverse effect level) or LOAEL (The lowest observed adverse effect level), recommendations of the FAO (Food and Agriculture Organization) and WHO (World Health Organization). In particular, RfD for Pb was equal to 0.0036 mg/kg/day [35,36]; for Cu—0.04 mg/kg/day [37,38].

The estimation of non-carcinogenic risks was made for three different groups of population: the first children group (1–6 years), the second children group (7–16 years), and the adult group (18–65 years). The DI was calculated referring to the norms of apple consumption, approved in Ukraine: the first and the second children groups of population—30.1 and 33.4 kg apples per person per year respectively (Resolution of the Cabinet of Ministers of Ukraine of 11 October 2016 No 780); the adult group—50 kg apples per person per year [39]. Thus, the norm of daily consumption of apples for the first and the second children groups was equal to 82.47 and 91.5 g/day respectively; for the group of adult population—137 g/day. The average BW indices for the first and the second children groups were equal to 17 and 45.2 kg respectively, and for the adult group—78.65 kg [40].

The carcinogenic risk (CR) was calculated by Formula (5):

(5) CR=EDI ×SF

where: 

CR—cancer risk,

EDI—estimated daily intake (Formula (4)),

SF—the slope factor used to estimate the probability of increased cancer incidence over a lifetime.

Of all the heavy metals studied, we chose Cr and Cd for assessing the carcinogenic risk, according to the IARC (IARC (International Agency for Research on Cancer) classification. The SF is: Cd—15, Cr—0.5 [41].

The Principal component analyses (PCA) were conducted using software «Statistica Base». The statistical analysis and graphical processing were performed using the software «Microsoft Excel 2019».

## 3. Results and Discussion

### 3.1. Statistical Analysis of the Distribution of Heavy Metals in Apple Samples

The research results demonstrate a considerable spatial differentiation of HM accumulation in the apple fruits, grown in different functional zones of Kharkiv city, Ukraine (Table 1).

The results of statistical analysis confirm a significant difference in the content of heavy metals in apple samples from different sampling sites. The values of the coefficient of variation range from 17.46% to 51.42% (Table 2).

Thus, the content of heavy metals in the studied apple samples does not agree with the normal distribution.

Using principal component analysis (PCA) we identified five factors, that explain some of the features of the distribution of heavy metals in the studied apple samples (Table 3). Factor 2 has the closest relationship with Cr and Cd, factor 3 with Cu, factor 4 with Cr, and factor 5 with Zn and Cu. A high value of the positive relationship (more than 0.5) is characteristic of factor 2 and Cr, factor 3 and Cu.

Based on the PCA results, we can assume that there is a relation between apple contamination and industrial activities. Cr and Cd are associated with Factor 2, while other elements have a negative relationship with this Factor. Such a relationship may be due to the influence of atmospheric pollution from mechanical engineering enterprises located in the study area. Factor 3, associated with Cu, is probably the metallurgical enterprises of the area.

In general, the results of the statistical analysis and PCA may indicate a predominantly anthropogenic nature of the formation of the chemical composition of the studied apple samples. This is confirmed by an abnormal distribution, high values of standard deviations and coefficients of variation—up to 51%, the absence of a close correlation between most factors and the content of heavy metals. Such geochemical features are typical for urban geosystems of the post-Soviet space and are due to the specific type of nature management, as well as local factors (contamination of the territory by industrial and household waste, emissions from vehicles or enterprises, etc.).

### 3.2. Spatial Features of the Accumulation of Heavy Metals in Apple Samples

**Figure 3:**
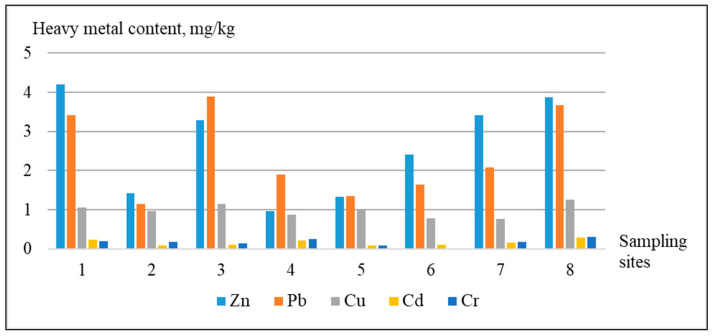
The content of heavy metals in the apple samples.

Consider in more detail the features of the accumulation of heavy metals in apple fruits grown in different functional zones of Kharkiv (Figure 3).

The Zn concentrations in the apple samples were from 4.19 mg/kg (garden in the private sector) to 0.98 mg/kg (square park). The content of Pb was the highest in the apple samples, grown on the school territory, household plot of a five-story apartment building, and private sector—above 3 mg/kg. The least value of the Pb content was observed in the apple samples of the transport area, i.e., 1.15 mg/kg. The highest concentrations of Cu were also particular for the apples of household plots of the five-story apartment building, school, and private sector, i.e., from 1.02 to 1.26 mg/kg. The least concentrations of Cu were marked in the apple samples grown in the territory of the transport area and industrial cluster—0.79 and 0.77 mg/kg respectively.

Among the studied HM, the least concentrations in the samples were observed concerning Cd (from 0.09 to 0.28 mg/kg) and Cr (from 0.02 to 0.32 mg/kg). The spatial distribution of Cr and Cd accumulation in the apple samples is in agreement with the regularities of distribution of other examined HM. The highest concentrations of Cd were fixed in the samples of the household plot—0.28 mg/kg; private sector—0.25 mg/kg; square park—0.21 mg/kg. Lower concentrations of Cd were found for the samples of the industrial cluster and transport area, i.e., 0.16 and 0.1–0.11 mg/kg respectively. The highest concentrations of Cr were also found in the samples of the household plot—0.32 mg/kg; square park—0.25 mg/kg; private sector—0.21 mg/kg. In the samples of other points, the Cr concentrations were less than 0.2 mg/kg.

The maximum and minimum content of Cd was found in the apple fruits, grown on the territory of household plots of five-story apartment buildings: sampling points № 8 and 5 respectively (Figure 1). The same situation was observed with all other studied HM. Particularly, in the sampling point No 8, the experiment fixed the maximum content of Cr, i.e., 0.32 mg/kg, whereas in the sampling point No 5, the content of Cr was equal to 0.1 mg/kg. The great difference of the HM content in the samples of those sampling points was probably caused by the atmospheric transfer from the industrial zone. The sampling point № 8 was located immediately near the industrial zone (almost 500 m), and the sampling point № 5, in the dormitory district.

The analysis of the HM distribution confirms the most intensive accumulation in the apples of the residential zone: household plots of residential buildings, square park, private sector. Such distribution is probably caused by soil pollution. While examining the soil cover, a considerable amount of anthropogenic inclusions, particularly batteries, plastic, packages, metal items, etc. was found. The distance from the land plot to the industrial objects is also of great importance. The apple sample from the sampling point, located on the territory of the industrial zone, was characterized by lower concentrations of HM than the sample from the sampling point at the distance of 500 m from the industrial zone. This is explained by the fact that pollutants from the flare of the industrial object settle on the surface at a definite distance, depending on the height of the flare device. Consequently, the components of the natural environment in the territory of the industrial object are less polluted, because they are located before the zone of pollutant settling.

It should be noted that the peculiarities of fruit contamination largely depend on the morphology of the plant. The apple tree, as a perennial plant, during its life period accumulates chemical elements arising through the root system from the soil [42]. The depth of the root system of an apple tree is on average 3–4 m, and the bulk of the roots is located at a depth of 1 m. Therefore, it is very likely that high concentrations of heavy metals were found in apple fruits of the residential zone due to the fact that the soils of these test sites (household plots of multi-storey buildings and other buildings) are contaminated with solid household and industrial waste. Visual inspection of the test sites revealed numerous artifacts in the soil cover: batteries, packaging made of iron and other materials, covers and other waste. The age of buildings in the residential zone is 40–60 years and waste in the adjacent soil cover has been accumulating for decades.

### 3.3. Estimation of the Safety for Human Health

The mentioned concentrations of HM in the studied samples of apples were compared with their maximum levels, set by the EC, FAO, and Ministry of Health of Ukraine (methodology of safety estimation and value of the maximum levels are presented above). In environmental science, the maximum values or threshold permissible concentrations of pollutants determine the amount of pollutant, which, under permanent or temporary contact with a person, does not have a negative impact on his/her health and does not cause negative consequences for future generations. It is worth noting that the maximum levels of chemical substances and elements in food products or components of the environment differ depending on the legal norms of any specific country.

**Figure 4:**
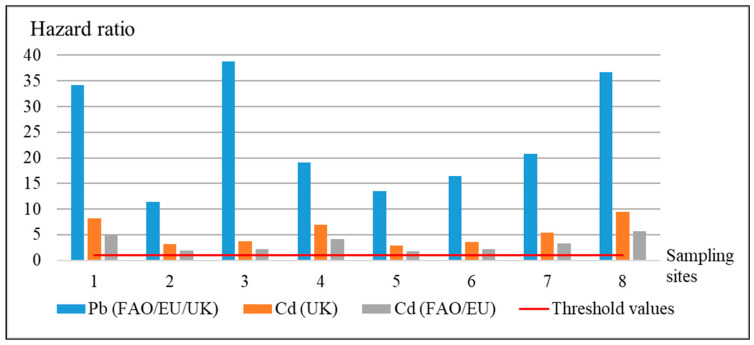
Estimation of the safety of apple consumption for human health by the hazard quotient.

The results of comparison of the actual concentrations of HM in the apple samples and their maximum levels confirm the presence of a potential hazard for human health (Figure 4). The samples from all sampling points were characterized by the excess of the maximum levels of pollutants: Pb from 11.47 to 38.86 times (according to the norms of the FAO, EU, Ministry of Health of Ukraine [27,30,31]); Cd—from 2.93 to 9.47 times (referring to the norms of the Ministry of Health of Ukraine [31]); Cd—from 1.76 to 5.68 times (referring to the norms of the FAO, EU [27,30]).

The highest values of the hazard quotient by Pb were particular for the sampling points № 1, 3, and 8 (from 34.21 to 38.86); by Cd, for the sampling points 1, 4, and 8 (from 4.2 to 5.68). All sampling points belong to the residential zone. The sampling point № 4, located on the territory of the square park can be also considered as belonging to the residential area. Nowadays, the square park is an abandoned territory with spontaneous planting of trees and grass, surrounded by residential complexes and objects of social and economic infrastructure (garage cooperative, motor way, kindergarten, etc.). As already mentioned, the highest levels of pollution of the samples of the residential area were probably caused by the considerable pollution of soil cover from household and industrial wastes.

### 3.4. Estimation of the Risks for Human Health

**Figure 5:**
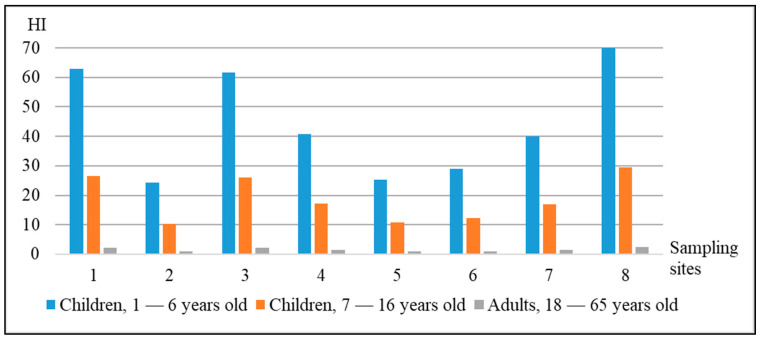
Estimation of the total non-carcinogenic risk for the groups of adult and children population.

Results of the research demonstrate an increase of the permissible level of the HI for all studied groups of population by the samples of apples from almost all sampling points (Figure 5). The exception is represented by the sample from the point № 2, the group of adult population of the age of 18–65 years (the HI level is equal to 0.88). A significant difference in the HI levels is observed between the groups of population of different age. The range of the values of the total non-carcinogenic risk for the children group of 1–6 years stays within the limits from 24.37 to 70.11 HI; for the children group of 7–16 years, from 10.28 to 29.59 HI; for the adult group of 18–65 years, from 0.88 to 2.53 HI.

**Figure 6:**
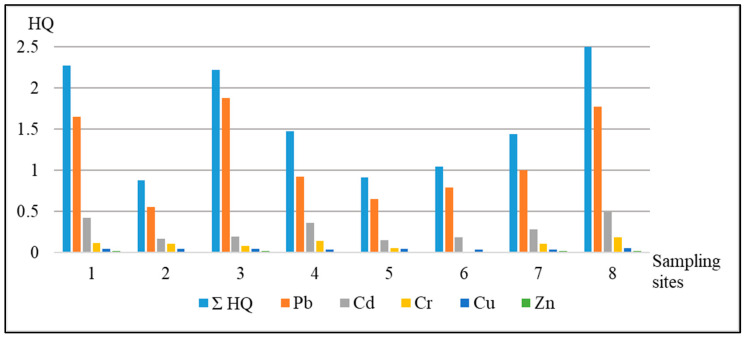
Estimation of the non-carcinogenic risk for the group of adult population.

The current research considers the HI level by each HM separately for the example of the group of adult population (Figure 6). The ranges of the values of non-carcinogenic risk are for Pb—0.55–1.78 HQ; Cd—0.15–0.49 HQ; Cr—0.01–0.18 HQ; Cu—0.03–0.05 HQ; Zn—0.01–0.02 HQ. Thus, the HQ levels separately for Cd, Cr, Cu, and Zn stay within the norms and are equal to less than 1. The HQ of Pb exceeds the norm in the samples from the sampling points № 1, 3, and 8 (residential area: private sector, school and household plot of the five-story apartment building respectively).

This work supplies the analysis of the probable harmful non-carcinogenic effects, which can appear due to the population’s consumption of polluted apples from the studied territory. According to the program EPA IRIS non-carcinogenic effects are determined for Cd and Zn under peroral intake from the food products. According to the IRIS, Cd critically influences the urino excretory system, causing heavy proteinuria, i.e., release of an excessive amount of protein with urine. Zn critically influences the immune system and blood-vascular system, causing reduction of the content of Cu and Zn-superoxide dismutase in erythrocytes [34].

In modern researches, there is information on the risks of other harmful non-carcinogenic effects, related to the excessive intake of Cd and Zn. In particular, the probable harmful effects of Cd impacting on the human body include development of the nephrotic syndrome, immune suppression, disorder of the endocrine system, etc. [43]. The results of modern researches confirm the relative safety of Zn as compared to other HM. It is noted, that high doses of Zn can cause problems of Cu absorption and thus, a considerable share of the toxic impact of Zn is related to Cu deficit [44].

The critical effects of the intake of an excessive doze of Pb, Cr, and Cu to the human body with food products have not been determined in the IRIS. Based on the analysis of literary sources, one can make conclusions on the development of such harmful non-carcinogenic effects, related to Pb, as an increase of protein in epithelial cells [34], an increase of the number of inflamed cells, as well as respiratory, cardio-vascular and neurological diseases [45]. The excessive intake of Cu to the human body can cause liver diseases and the onset of serious neurological defects [46] together with disorder of immune functions [47]. The toxic effect of Cr for the human body is related with the risk of disorders of liver, kidneys, and stomach, etc. [48].

The results of the carcinogenic risk assessment are presented in Table 4.

The acceptable level of carcinogenic risk is the range of CR values from 10^−6^ to <10E^−4^ [49]. The results of the study showed that the total carcinogenic risk of eating apples from all studied sampling sites exceeds the permissible level and is 10^−3^. If we consider the carcinogenic risk separately for each element, then the Cr content is in a safe range—from 10^−4^ to 10^−5^, whereas the content of Cd in apples from all studied sampling sites is a carcinogenic hazard, because it is 10^−3^. Thus, Cd has the greatest contribution in the formation of the total carcinogenic risk of eating apples.

Based on the above, we can conclude that the intake of the excessive concentrations of the studied HM (Pb, Cr, Zn, Cu, Cd) with food products into the human body causes risks of development of harmful non-carcinogenic effects in the endocrine, cardio-vascular, and nervous systems, liver and kidneys, as well as disorders of the immune system, etc.

## 4. Conclusions

The rapid development of UA produces challenges, among which the leading position is occupied by the issue of quality and safety of vegetable products, grown within the urban environment. Modern urban geosystems are characterized by a complexity and diversity of anthropogenic geochemical flows, which create zones of increased ecological hazard. The results of scientific researches in different countries confirm an increase of the maximum levels of pollutants in urban vegetable products up to by ten fold.

The chemical content of crops of the urban geosystems significantly differs in different regions of the world depending on the geo-ecological conditions of the territory. The research, described in the article, was conducted on the territory of the Ukrainian city of Kharkiv, i.e., one of the largest cities of the post-Soviet area. The urban geosystem of that region is characterized by the close neighborhood of anthropogenic places with residential and recreational zones—the availability of a considerable area, free from buildings; traditions of growing vegetable products everywhere, particularly in household plots of residential complexes and the industrial clusters. The post-Soviet cities possess a significant natural-climatic and land potential for UA development on the one hand, and on the other hand serve as sources for formation of increased levels of ecological hazards.

The results of the conducted research confirm that the content of HM in the apple fruits from all sampling points in Kharkiv city, Ukraine, are over the safe norms, particularly Pb—from 11.47 to 38.86 times (norms of the FAO, EU, and the Ministry of Health of Ukraine [27,30,31]); Cd—from 2.93 to 9.47 times (norms of the Ministry of Health of Ukraine [31]); Cd—from 1.76 to 5.68 times (norms of the FAO and EU [27,30]). The greatest excess of the maximum levels of HM was found in the samples of apples from the sampling points of the residential area, particularly the private sector, the household plots of multi-story apartment building, and the square park. Such distribution was probably caused by pollution of the land plots of the residential area by household and industrial wastes, like batteries, plastic, metal items, etc.

The HI levels differ depending on the age content of the studied groups: from 24.37 to 70.11 HI (children group, 1–6 years); from 10.28 to 29.59 HI (children group, 7–16 years); from 0.88 to 2.53 HI (adult group, 18–65 years). The risks of development of non-carcinogenic effects can be related to the disorders of the immune system, blood, urino excretory and nervous systems, as well as problems of the normal functioning of liver and kidneys.

The total carcinogenic risk, calculated on the basis of the content of Cr and Cd in apple samples, exceeds the permissible level and is 10^−3^. The greatest contribution to the formation of the total carcinogenic risk is made by Cd.

To sum up, development of UA within the boundaries of the post-Soviet countries needs a complex investigation of the ecological conditions of urban environments with ranging of the land plots by the level of suitability for crop growing. Particular attentions should be paid to informing the population on the safety of consumption of vegetable products, grown in the city gardens and the solution of the problem of urban environment pollution, particularly within the framework of improvement of the ecological culture of the population.

## Figures and Tables

**Table 1:** The content of heavy metals in the apple samples.

Heavy Metals	Heavy Metal Content in Apple Samples. mg/kg							
	1	2	3	4	5	6	7	8
Cr	0.206	0.182	0.144	0.246	0.096	0.0225	0.182	0.315
Zn	4.192	1.431	3.281	0.964	1.324	2.404	3.417	3.881
Cu	1.067	0.966	1.142	0.882	1.024	0.789	0.766	1.264
Cd	0.245	0.097	0.11	0.21	0.088	0.108	0.162	0.284
Pb	3.421	1.147	3.886	1.904	1.347	1.642	2.075	3.672

**Table 2:** The standard deviation of the content of heavy metals in samples of apples.

Heavy Metals	Valid N	Mean	Minimum	Maximum	St. Dev.	Coefficient of Variation
Cr	8	0.17419	0.0225	0.315	0.089565	51.42%
Zn	8	2.61175	0.964	4.192	1.254433	48.03%
Cu	8	0.9875	0.766	1.264	0.172392	17.46%
Cd	8	0.163	0.088	0.284	0.075014	46.02%
Pb	8	2.38675	1.147	3.886	1.100145	46.09%

**Table 3:** The results of the Principal Components Analysis.

Variable	Factor Coordinates of the Variables. Based on Correlation				
	Factor 1	Factor 2	Factor 3	Factor 4	Factor 5
Cr	−0.760731	0.611470	0.023584	0.210921	0.048464
Zn	−0.758606	−0.503451	−0.366144	0.046105	0.186729
Cu	−0.744405	−0.099907	0.645333	−0.096266	0.100784
Cd	−0.839401	0.381767	−0.309361	−0.228498	−0.041766
Pb	−0.892219	−0.369111	0.043831	0.076251	−0.244881

**Table 4:** The results of the carcinogenic risk assessment.

CR	Sampling Sites							
	1	2	3	4	5	6	7	8
Cd	7.19 × 10^−3^	2.85 × 10^−3^	3.23 × 10^−3^	6.17 × 10^−3^	2.58 × 10^−3^	3.17 × 10^−3^	4.76 × 10^−3^	8.34 × 10^−3^
Cr	2.02 × 10^−4^	1.78 × 10^−4^	1.41 × 10^−4^	2.41 × 10^−4^	9.39 × 10^−5^	2.20 × 10^−5^	1.78 × 10^−4^	3.08 × 10^−4^
Σ	7.39 × 10^−3^	3.03 × 10^−3^	3.37 × 10^−3^	6.41 × 10^−3^	2.68 × 10^−3^	3.19 × 10^−3^	4.93 × 10^−3^	8.65 × 10^−3^

## Data Availability

Not applicable.
